# CO_2_ complexation with cyclodextrins

**DOI:** 10.3762/bjoc.19.78

**Published:** 2023-07-17

**Authors:** Cecilie Høgfeldt Jessen, Jesper Bendix, Theis Brock Nannestad, Heloisa Bordallo, Martin Jæger Pedersen, Christian Marcus Pedersen, Mikael Bols

**Affiliations:** 1 Department of Chemistry & Niels Bohr Institute, University of Copenhagen, 2100 København Ø, Denmark

**Keywords:** carbon dioxide, crystals, cyclodextrin, gas binding

## Abstract

Carbon dioxide (CO_2_) emissions from industrial processes, power generation, and transportation contribute significantly to global warming and climate change. Carbon capture and storage (CCS) technologies are essential to reduce these emissions and mitigate the effects of climate change. Cyclodextrins (CDs), cyclic oligosaccharides, are studied as potential CO_2_ capture agents due to their unique molecular structures and high selectivity towards CO_2_. In this paper we have investigated binding efficiency of a number of cyclodextrins towards CO_2_. It is found that the crystal structure of α-cyclodextrin with CO_2_ has a 1:1 stoichioimetry and that a number of simple and modified cyclodextrins bind CO_2_ in water with a *K*_g_ of 0.18–1.2 bar^−1^ (7–35 M^−1^) with per-*O*-methyl α-cyclodextrin having the highest CO_2_ affinity.

## Introduction

The concentration of carbon dioxide (CO_2_) in the Earth's atmosphere has increased significantly in recent decades [1–2] presumably due to human activity and the extensive burning of fossil fuels. With the ability of CO_2_ to absorb energy from sunlight [3], global warming and climate change is expected and serious consequences anticipated. To mitigate the effects of climate change, it is essential to reduce CO_2_ emissions from various sources, such as industrial processes, power generation, and transportation. Carbon capture and storage (CCS) technologies are crucial to achieving this goal. CCS technologies involve capturing CO_2_ from industrial processes or power plants, transporting it to a storage site, and storing it underground or consuming it by forming polymers [4] or fuels from CO_2_ [5–6]. However, the implementation of CCS technologies faces many challenges, including high costs, energy consumption, and the need for large-scale infrastructure. One of the characteristics of carbon capture technologies that use amines is the formation of a covalent bond to the CO_2_ molecule. This bond obviously has to be broken in order to regenerate the material with resulting energy cost [7]. It is therefore logical to explore capture alternatives where CO_2_ is captured by non-covalent binding.

**Figure 1:**
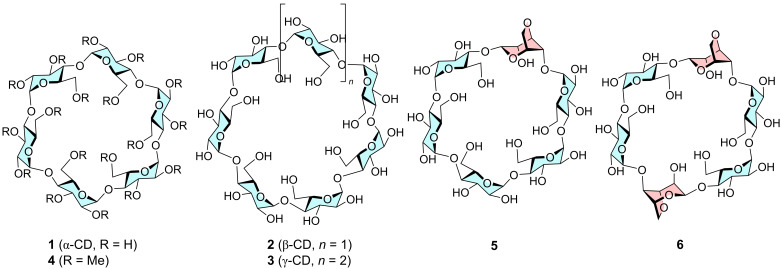
Structure of cyclodextrins **1**–**6** studied in this work.

Henglein and Cramer showed many years ago that α-cyclodextrin (**1**, Figure 1), when treated with CO_2_ under pressure for several days gave crystals with the gas trapped inside [8]. According to Cramer only **1** was able to form crystals, while larger cyclodextrins such as **2** and **3** and did not. Recently the solid complex of CO_2_ and **1** have been studied as a food product additive [9–11]. Anions of **1** in DMSO have been found to have a high capacity for capturing of CO_2_ [12]. Being cheap, biodegradable and eco-friendly these carbohydrates might form the basis in an economic CO_2_ capture technology. We have therefore studied the binding of CO_2_ to simple cyclodextrins to determine binding stoichiometry and affinity. It is found that the crystal structure of α-cyclodextrin with CO_2_ has 1:1 stoichioimetry and that a number of simple and modified cyclodextrins bind CO_2_ in water with a *K*_g_ of 0.18–1.2 bar^−1^ (7–35 M^−1^).

## Results and Discussion

**Figure 2:**
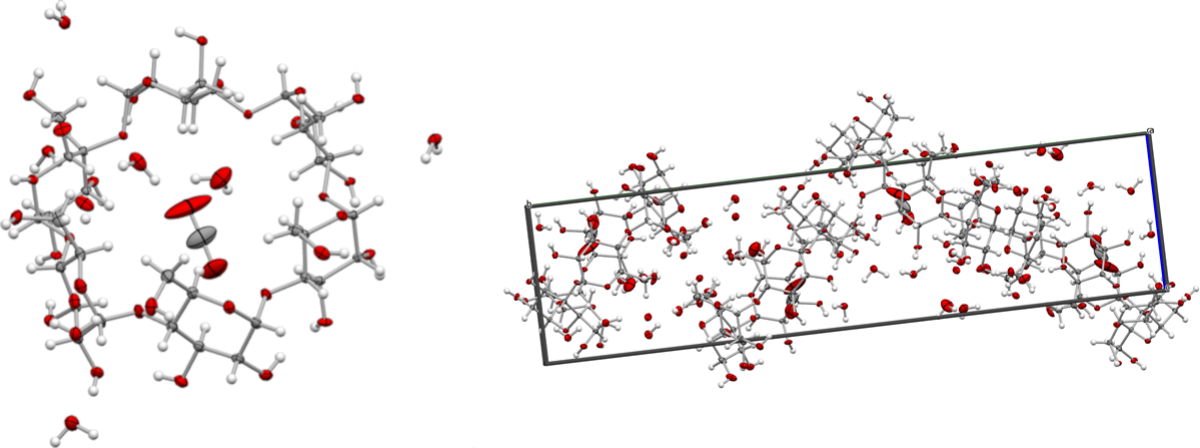
X-ray crystal structure of CO_2_ bound to α-CD.

When a saturated solution of **1** was treated with CO_2_ at a pressure from 6–20 bar in an autoclave for 1–19 days, crystals containing CO_2_ were obtained in 16–63% yield (Table 1). The most important factor for getting higher yields was sufficient time for the crystallization, while the pressure was found less important. From the binding constant measured below and the 1:1 stoichiometry (*K*_g_ = 0.18 bar^−1^, Table 2) we know that 1/2 to 3/4 of the cyclodextrin is filled with CO_2_ at the pressures used as shown in the 5th column of Table 1. As CO_2_ is in large excess high crystal yields can be obtained even though the CD cavity is only partially filled, because more CO_2_ is bound in solution as crystallization proceeds. When equilibrium is reached as in entries 1–3 (Table 1) the concentration of **1**·CO_2_ is 0.04 M which must be the solubility of this complex in water. X-ray crystallography of the crystals showed a 1:1 complex of CO_2_ to α-CD with CO_2_ bound in the center of the wide, secondary rim of the α-CD cavity (Figure 2). Two of the hydroxymethyl side groups on the primary narrow rim are disordered. The disordering was modeled over two positions for each hydroxymethyl group with one of the positions leading to engagement in hydrogen bonding to water molecules bound at the narrow rim with a combined occupancy of 0.75. Additionally, five fully occupied water molecules are found in the structure one of which is best modeled as split over two positions yielding in total 5.75 mol of water per CO_2_. The hydration is similar to that of native α-CD [13] and that of the krypton inclusion complex which has 5.28 water/Kr [14]. The CO_2_ molecule refines with an optimal occupancy of 0.84 and linear geometry (178.2(6)^o^) with C–O bond lengths of 1.138(7) Å/1.146(5) Å. Refinement with full occupancy is also consistent with the diffraction data and yields realistic geometries.

**Table 1:** The results of crystallization of α-cyclodextrin from water in an atmosphere of CO_2_ carried out in a pressure autoclave. [CD]_tot_ is the starting concentration of cyclodextrin. [CD·CO_2_]/[CD]_tot_ is the calculated ratio of bound CO_2_ in solution using a *K*_g_ of 0.18 bar^−1^.

Entry	CD	[CD]_tot_ (M)	Pressure (bar)	[CD·CO_2_]/[CD]_tot_	Time (days)	Crystal yield

1	**1**	0.15	6	52%	19	48%
2	**1**	0.15	7	56%	3	50%
3	**1**	0.15	10	64%	2	63%
4	**1**	0.15	17	75%	1	17%
5	**1**	0.15	20	78%	1	16%

When the crystals were treated with water, CO_2_ was released as bubbles as the crystals were dissolving, which was also very clearly observed under a microscope.

**Figure 3:**
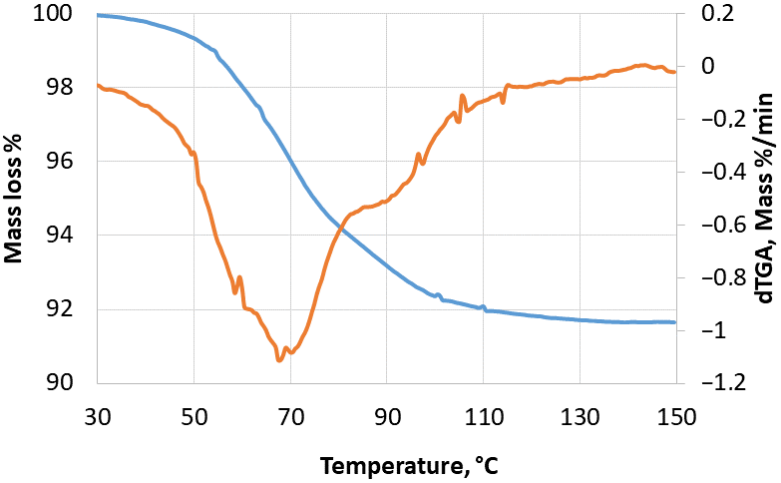
TGA curve (blue) and dTGA curve (red) for CO_2_-**1** crystals. Two lumps are seen with the former predominantly being CO_2_ and the second pre-dominantly water.

To get more information about the CO_2_ content in the crystal samples we also analyzed the crystals by thermogravimetric analysis. The crystal samples where heated to 26–200 °C at different rates and weight loss observed while the gas release was monitored by IR spectroscopy. Two distinguished weight decrease steps were seen in the TGA curve and very evident from the dTGA curve (Figure 3). The first weight decrease was seen around 50–75 °C and accounted for 5–6%, while the second weight decrease step normally was observed at 75–100 °C and accounte for 2–3%. IR analysis of the gas outlet showed both the characteristic water absorption at 3200–3600 cm^−1^ and the C=O band of CO_2_ at 2350 cm^−1^ during the both weigth losses but mainly CO_2_ at the first lump and predominantly water at the second loss. This also suggest a comparatively weak binding of the CO_2_.

**Figure 4:**
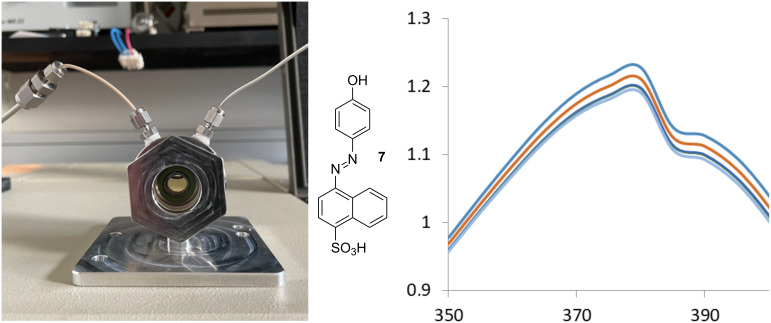
Cell used to measure vis spectra under pressure (left), structure of **7** (middle) and spectrum of **7** (40 μM) and **1** (2 mM) in citrate phospate buffer pH 3 (right) from 350–400 nm with 0 (blue), 2 (red), 4 (green), 6 (orange) and 8 (grey) bar of CO_2_.

**Figure 5:**
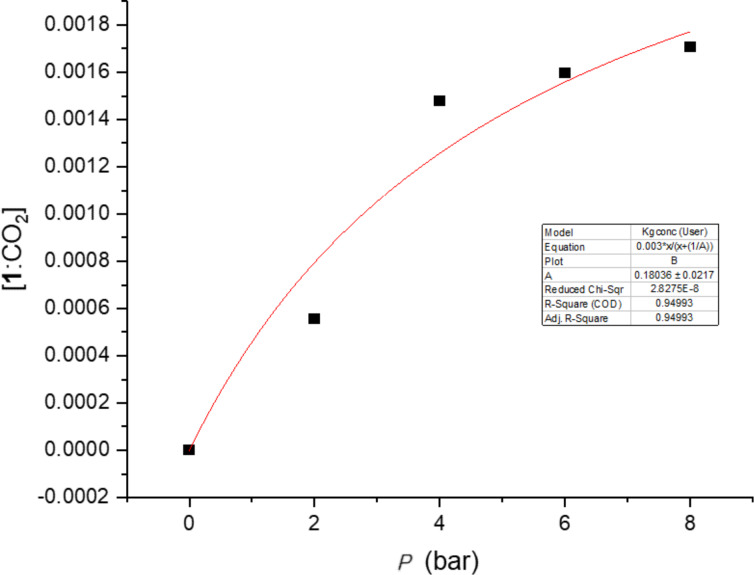
Binding of CO_2_ to **1** as a function of pressure.

We determined the binding constant for CO_2_ to cyclodextrins using a pressure cell and a UV–vis competition assay with an azo-dye (4-((4-hydroxyphenyl)azo)-1-naphthalenesulfonic acid (**7**) Figure 4) [15] as an indicator. The UV–vis spectrum of **7** changes on binding to cyclodextrins and we can thereby indirectly monitor the binding of CO_2_ to the CD by observing the change in spectrum of **7** provided **7** and CO_2_ compete for the binding site. To avoid problems with formation of hydrogencarbonate the experiments were conducted in a buffer at pH 3 where only a minor fraction of the carbonic acid (p*K*_a1_ = 3.6) is dissociated and since the hydration constant of CO_2_ is small (1.7 × 10^–3^) more than 99% of CO_2_ in solution is the dissolved gas at this pH. First the dissociation constants of **7**–CD complexes at pH 3, were determined (Table 2). When a solution of **7** and excess cyclodextrin was subjected to a CO_2_ atmosphere at 2–8 bar in the pressure cell this gave, after equilibration for 2 hours, a change in the vis spectrum (Figure 4). The change was consistent with the displacement of **7** from the cavity by CO_2_ and change in the amount of azodye was used to calculate the amount of CO_2_ bound to the cyclodextrin. From the change in absorption at 370 nm as compared to the reference spectrum without cyclodextrin, we determined the gas binding constants from non-linear regression of bound CO_2_ versus CO_2_ pressure as shown for **1** in Figure 5. This gave the gas binding constant *K*_g_ = [CD·CO_2_]/[CD]P_CO2_ given in Table 2 in bar^−1^. This constant gives the fraction of CD bound CO_2_ in water under a partial pressure of CO_2_. Using a literature value of the solubility of CO_2_ in water at 1 bar (34 mM) and Henrys law the more traditional aqueous binding constant *K*_a_ = [CD·CO_2_]/[CD][CO_2_] in molar terms could be calculated (Table 2). For α-cyclodextrin (**1**) a *K*_g_ of 0.18 bar^−1^ was obtained – a small value, which means that even at a partial pressure of 5 bar less than 1/2 the CD cavity is filled. The rationale for the poor binding is probably the large discrepancy between the size of CO_2_ and the CD cavity. **1** has a cavity of 174 Å^3^ while CO_2_ is a very small molecule with kinetic diameter of 3.3 Å and a spherical volume of only 19 Å^3^.

**Table 2:** *K*_d_ for binding of **7** and *K*_a_ for binding CO_2_ for cyclodextrin derivatives in citrate-phosphate buffer at pH 3.

CD	Residues^a^	*K*_d_ of 7 at pH 3 (M)	*K*_g_ for CO_2_ (bar^−1^)	*K*_a_ for CO_2_ (M^−1^)^b^	Cavity volume^c^

**1** (α-CD)	6	4.51 (±1.42) × 10^−4^	0.18 ± 0.02	5.3 ± 0.6	174 Å^3^
**2** (β-CD)	7	7.36 (±3.16) × 10^−4^	no binding	no binding	262 Å^3^
**3** (γ-CD)	8	1.63 (±1.3) × 10^−4^	no binding	no binding	427 Å^3^
**4**	6	1.33 (±0.31) × 10^−4^	1.2 ± 0.14	35 ± 4.1	174 Å^3^
**5**	6	2.25 (±0.11) × 10^−3^	0.75 ± 0.08	22 ± 2.4	155 Å^3^
**6**	6	1.39 (±0.25) × 10^−3^	0.34 ± 0.06	9.8 ± 1.8	115 Å^3^

^a^Number of monosaccharide residues. ^b^Calculated from the value in bar^−1^ by dividing with solubility of CO_2_ at one bar. ^c^Calculated as described by Szejtli [18].

The binding for **2** and **3** and for the three derivatives of **1**, **4**, **5** and **6** were also investigated and the binding curves for determination of *K*_g_ for these derivatives are shown in Supporting Information File 1 (Figures S1–S3). The rationale for these compounds were the following:

- β- (**2**) and γ-cyclodextrins (**3**) were also studied by Cramer, but gave no crystals although they might still bind CO_2_.
- Compound **4** (permethylated α-cyclodextrin) [16] is in terms of hydrogen bonding properties and polarity vastly different from **1** yet still water-soluble.
- Compounds **5** and **6** containing 3,6-anhydrides in the α-cyclodextrin structure were chosen because they have a smaller cavity according to modelling. In models the diameter of **5** was measured and it was 5.0 Å and that of **6** was 4.6 Å with an α-CD height of 7.9 Å to give the cavity volumes given in Table 2. These compounds were made by tosylation of the corresponding partially benzylated cyclodextrins, hydrogenolysis and base treatment (see Supporting Information File 1 for experimental details). Compounds **5** and **6** have previously been made by direct tosylation of α-cyclodextrin which is a shorter route [17]. However, in our hands the direct tosylations were difficult to handle and the protection–deprotection route proved a more reliable route to pure compounds.

For **2** and **3** we found no binding which is in line with the absence of gas-crystals from these cyclodextrins. The lack of binding must, in part, be linked to the very large cavities of these molecules (Table 2). The anhydrocyclodextrins **5** and **6** have a slightly stronger binding than **1**, which on the other hand is due to these molecules having smaller cavities, although the less modified and larger monoanhydro derivative **5** was the stronger binder revealing that other factors are important. The permethylated cyclodextrin **4** was found to be the best CO_2_ binder, which presumably is related to its hydrophobic character. Compound **4** is known to bind little water in the crystal (1 or 0 molecules) and probably also in solution as witnessed by its reverse temperature dependence on solubility in water [16]. Therefore, binding of the unipolar CO_2_ molecule is expected to cause less water H-bond disruption in this host.

## Conclusion

This work shows that CO_2_ is bound 1:1 by α-cyclodextrins and that the affinity can be improved with a smaller cavity and more lipophilic cyclodextrin derivative. It suggests that stronger CO_2_ binders can be found by improving on these two traits.

## Experimental

**Crystallization experiments.** A cyclodextrin solution was prepared by dissolving **1** in Milli-Q water to the indicated concentration (Table 1) . The solution was then filtered, placed in an autoclave and pressurized with CO_2_ gas (pressure 6–20 bar) at 25 °C for 1–17 days. The resulting crystals were collected and filtered by suction filtration. The crystals were left to dry in a desiccator at normal pressure over CaCl_2_.

**X-ray analysis.** The x-ray crystallographic studies were carried out on single crystals, which were coated with mineral oil, mounted on kapton loops, and transferred to the nitrogen cold stream of the diffractometer. The single-crystal X-ray diffraction studies were performed at 100(2) K on a Bruker D8 VENTURE diffractometer equipped with a Mo Kα high-brilliance IμS radiation source (λ = 0.71073 Å), a multilayer X-ray mirror and a PHOTON 100 CMOS detector, and an Oxford Cryosystems low temperature device. The instrument was controlled with the APEX3 software package using SAINT (Bruker; Bruker AXS, Inc. SAINT, Version 7.68A; Bruker AXS: Madison, WI, 2009). Final cell constants were obtained from least squares fits of several thousand strong reflections. Intensity data were corrected for absorption using intensities of redundant reflections with the program SADABS (Sheldrick, G. version 2008/2; University of Göttingen: Germany, 2003). The structures were solved in Olex2 using SHELXT and refined using SHELXL (Table 3) [19]. There is some disorder in one water molecule, which was modelled over two positions and some disorder in at least two of the primary alcohol groups, which was modelled. The latter disorder is not uncommon in α-cyclodextrin structures [20]. In the reported structure, CO_2_ was refined with an occupancy of 0.84.

**Table 3:** Crystal data and structure refinement for α-CD•CO_2_•5.75 H_2_O.

CCDC deposition number	CCDC 2266629
empirical formula	C_36.84_H_69.45_O_37.42_
formula weight	1113.90
*T*/K	100(2)
crystal system	orthorhombic
space group	*P*2_1_2_1_2_1_
*a*/Å	9.3721(6)
*b*/Å	14.3361(10)
*c*/Å	37.100(2)
α/°	90
β/°	90
γ/°	90
volume/Å^3^	4984.8(6)
Z	4
ρ_calc_g/cm^3^	1.484
μ/mm^-1^	0.135
F(000)	2365.0
crystal size/mm^3^	0.244 × 0.42 × 0.28
radiation	Mo Kα (λ = 0.71073)
2Θ range for data collection/°	4.35 to 61.996
index ranges	−13 ≤ h ≤ 13, −20 ≤ k ≤ 20, −47 ≤ l ≤ 53
reflections collected	76519
independent reflections	15881 [*R*_int_ = 0.0370, *R*_sigma_ = 0.0321]
data/restraints/parameters	15881/20/758
goodness-of-fit on F^2^	0.993
final R indexes [*I*>=2σ (*I*)]	*R*_1_ = 0.0396, *wR*_2_ = 0.0966
final R indexes [all data]	*R*_1_ = 0.0477, *wR*_2_ = 0.1016
largest diff. peak/hole / e Å^−3^	0.84/-0.53
Flack parameter	0.16(15)

**TGA measurements.** These analyses were carried out on a Netzsch TG 209 F1 Libra fitted with a FTIR detector from Bruker. Samples were prepared by crushing the crystals before putting them in an aluminium crucible ensuring full coverage of the bottom. The analysis shown in Figure 3 was started at 28 °C, and was finished at a temperature of 150 °C with a heating rate of 5 °C/min.

**Determination of dissociation constants between 7 and CD’s.** Samples were prepared that consisted of citrate-phosphate buffer (pH 3, 50 mM), **7** (40 μM) and an increasing concentration of cyclodextrin (**1**–**6**; [CD] = 0 or 1.3–22 mM). For each sample the spectrum was recorded from λ = 350–600 nm. For each cyclodextrin a change in the vis spectrum of **7** was seen. From Benesi–Hildebrand plots at 380 nm the *K*_d_ values given in Table 2 were found.

**Determination of association constants between CD’s and CO_2_.** Samples were prepared that consisted of citrate-phosphate buffer (pH 3, 50 mM), **7** (40 μM) and a fixed concentration of cyclodextrin ([CD]_o_; 1–4 mM) and put into the pressure cell. The spectrum was recorded at 0, 2, 4 , 6 and/or 8 bar CO_2_ pressure over the liquid after a 2–4 hour gas–liquid equilibration period. The *K*_g_ was determined as follows: [CD(**7**)] (and [**7**]) was calculated at each pressure from the change in absorption at 370 nm. From this [CD] was determined from the equation *K*_d_ = [CD][**7**]/[CD(**7**)] and [CD(CO_2_)] was calculated as CD_tot_ − [CD] − [CD(**7**)]. Now *K*_g_ was calculated by non-linear regression of [CD(CO_2_)] vs *P*_CO2_ as following the equation [CD(CO_2_)] = [CD]_o_*P*_CO2_/(*P*_CO2_+(1/*K*_g_)) as shown for **1** in Figure 5 and for **4**–**6** in Supporting Information File 1 (Figures S1–S3). This gave the *K*_g_ values in Table 2.

## Supporting Information

File 1 Experimental and analytical data.
