# Supplementary material for: CO2 complexation with cyclodextrins
Source: Beilstein J Org Chem. 2023 Jul 17;19:1021–7. doi: 10.3762/bjoc.19.78 (PMC10366446; doi:10.3762/bjoc.19.78)

BEILSTEIN JOURNAL OF ORGANIC CHEMISTRY

Supporting Information

for

CO_2 complexation with cyclodextrins

Cecilie Høgfeldt Jessen, Jesper Bendix, Theis Brock Nannestad, Heloisa Bordallo, Martin Jæger Pedersen, Christian Marcus Pedersen and Mikael Bols

Beilstein J. Org. Chem. **2023,** *19,* 1021–1027. doi:10.3762/bjoc.19.78

Experimental and analytical data

License and Terms: This is a supporting information file under the terms of the Creative Commons Attribution License (https://creativecommons.org/licenses/by/4.0). Please note that the reuse, redistribution and reproduction in particular requires that the author(s) and source are credited and that individual graphics may be subject to special legal provisions.
The license is subject to the *Beilstein Journal of Organic Chemistry* terms and conditions: (https://www.beilstein-journals.org/bjoc/terms)

Contents

Procedure and reaction scheme for synthesis of **5**	S1
Procedure and reaction scheme for synthesis of **6**	S3
Plots of Complex formation vs CO_2 pressure (Figures S1–S3)	S5
Specification of pressure cell	S7
References	S7
800 MHz NMR spectra of **5–6**	S8–19

Experimental

General. NMR assignment of **5** and **6** were done based on elucidation of HSQC, ROESY, COSY and TOCSY spectra carried out on an 800 MHz spectrometer at the cOpenNMR, an infrastructure facility funded by the Novo Nordisk Foundation (#NNF18OC0032996). In case of **5** only partial elucidation was possible and a number of signals are assigned to number of possible residues i.e. if a signal could be assigned to the D or E ring is marked D/E. In assigning the glucose residues the chemically different or altered residues are labelled A (and/or D) and the other residues labelled from that residue in opposite direction of glycosylation i.e. B is the residue that is glycosylated on 4-O of residue A and so on. All chemicals used were bought at chemical suppliers in reagent purity (98%>) and used as such.

Synthesis of 3^A,6^A-anhydro-α-cyclodextrin (5). A solution of 2^{A-F},3^{A-F},6^{B-F}-heptadecakis-*O*-benzyl-*α*-cyclodextrin [1] (0.650 g, 0.269 mmol) and *p*-toluenesulfonyl chloride (0.497 g, 2.61 mmol) in dry pyridine (9 mL) was stirred for 20 h under nitrogen atmosphere at room temperature. The reaction was quenched with water (10 mL) and CH_2Cl_2 (10 mL). The organic phase was washed with water (2 × 10 mL), dried using $MgSO_4$ and filtered. Evaporation in vacuo removed the solvents and resulted in a yellow brown syrup as the crude product. The crude product was purified with flash column chromatography (heptane/EtOAc, 0→100% EtOAc) yielding the desired product as a yellow-brown syrup (239 mg, 89.9 μmol, 33 %). R_f (heptane/EtOAc 3:1) = 0.29.

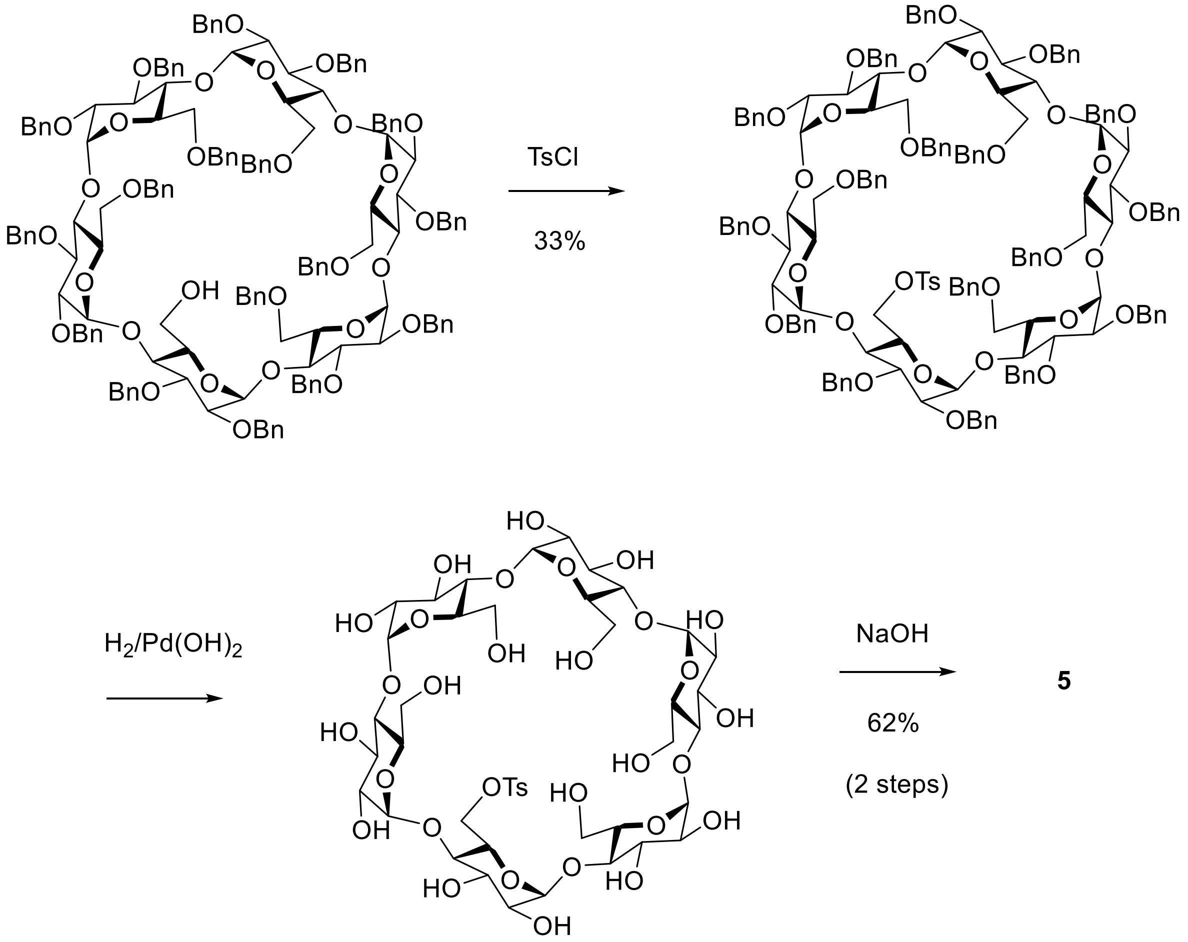

6^{A}-*O*-(p-Toluenesulfonyl)-2$^{A\text{-}F}$,3$^{A\text{-}F}$,6$^{B\text{-}F}$-heptadecakis-*O*-benzyl-α-cyclodextrin (239 mg, 89.9 μmol) was mixed with MeOH (15 mL) and EtOAc (15 mL) together with 1 M HCl (1 drop). Pearlman's catalyst, $Pd(OH)_2/C$ (73 mg, 0.520 mmol) was added to the reaction mixture. The reaction was stirred under hydrogen atmosphere for 2.5 h after which water (3 mL) was added to prevent precipitation of the product and the reaction was stirred for another 17.5 h. Filtration of the reaction mixture through a pad of celite yielded the 6^{A}-*O*-(p-toluenesulfonyl)-α-cyclodextrin. After removing the solvents in vacuo, the compound was dissolved in 1 M NaOH (2 mL) and stirred for 18 h at 40 °C. The solution was neutralized with addition of 1 M HCl, followed by filtration and removal of solvent in vacuo to yield the crude product. The crude product was purified by mixed bed ion chromatography, Dowex Marathon MR-3 (3 mL, H_2O) to afford the desired product **5** as a white foam (53 mg, 55.5 μmol, 62%). ^{1}H NMR (800 MHz, D_2O) δ 5.18 (d, *J* = 2.7 Hz, 1H, H-1^{A}), 5.16 (d, *J* = 3.9 Hz, 1H, H-1$^{D/F}$), 5.10 (d, *J* = 3.5 Hz, 1H, H-1$^{D/E}$), 5.09 (d, *J* = 4.1 Hz, 1H, H-1^{B}), 5.06 (d, *J* = 3.6Hz, 1H, H-1$^{C/E/F}$), 5.05 (d, *J* = 3.3 Hz, 1H, H-1$^{C/E/F}$), 4.61 (t, *J* = 2.7 Hz, 1h, H-5^{A}), 4.53 (t, *J* = 5.1 Hz, 1H, H-3^{A}), 4.28 (d, *J* = 11.0 Hz, 1H, H-6^{A}), 4.18 (dd, *J* = 5.4, 2.5 Hz, 1H, H-4^{A}), 4.09–3.84 (m, 23H, H-6b^{A}, H-3$^{B\text{-}F}$, H-4^{B,D}, H-5$^{B\text{-}F}$, H-6ab$^{B\text{-}F}$), 3.70-3.57 (m, 8H, H-4^{C,E,F}, H-2$^{B\text{-}F}$) ppm.

^{13}C NMR (201 MHz, D_2O) δ 101.9 (C-1$^{D/F}$), 101.6 (C-1$^{D/E}$), 101.2 (C-1^{B}), 100.3 (C-1^{A}), 100.2 (C-1$^{C/E/F}$), 100.1 (C-1$^{C/E/F}$), 80.9 (C-4$^{C/E/F}$), 80.7 (C-4$^{D/F}$), 80.7 (C-4$^{D/E}$), 80.6 (C-4$^{C/E/F}$), 80.4 (C-4^{B}), 79.6 (C-4^{A}), 74.3 (C-5^{A}), 73.1(C-5$^{C/E/F}$), 73.1 (C-3$^{C/E/F}$), 73.0 (C-3$^{D/E}$), 73.0 (C-5$^{C/E/F}$), 73.0 (C-6$^{C/E/F}$), 72.6 (C-5$^{D/E}$), 72.3 (C-5$^{D/F}$), 72.1 (C-5^{B}), 72.0

(C-2$^{C/E/F}$), 71.7 (C-3$^{C/E/F}$), 71.4 (2C, C-3$^{C/D/E/F}$), 71.4 (C-2^{B}), 71.3 (C-3^{A}), 71.3 (C-3^{B}), 71.3 (C-3$^{D/F}$), 70.9 (C-2$^{D/F}$), 69.0 (C-6^{A}), 68.7 (C-2^{A}), 60.8 (C-6$^{C/E/F}$), 60.8 (C-6$^{D/E}$), 60.3 (C-6$^{C/E/F}$), 60.2 (C-6$^{D/F}$), 60.1 (C-6^{B}) ppm.

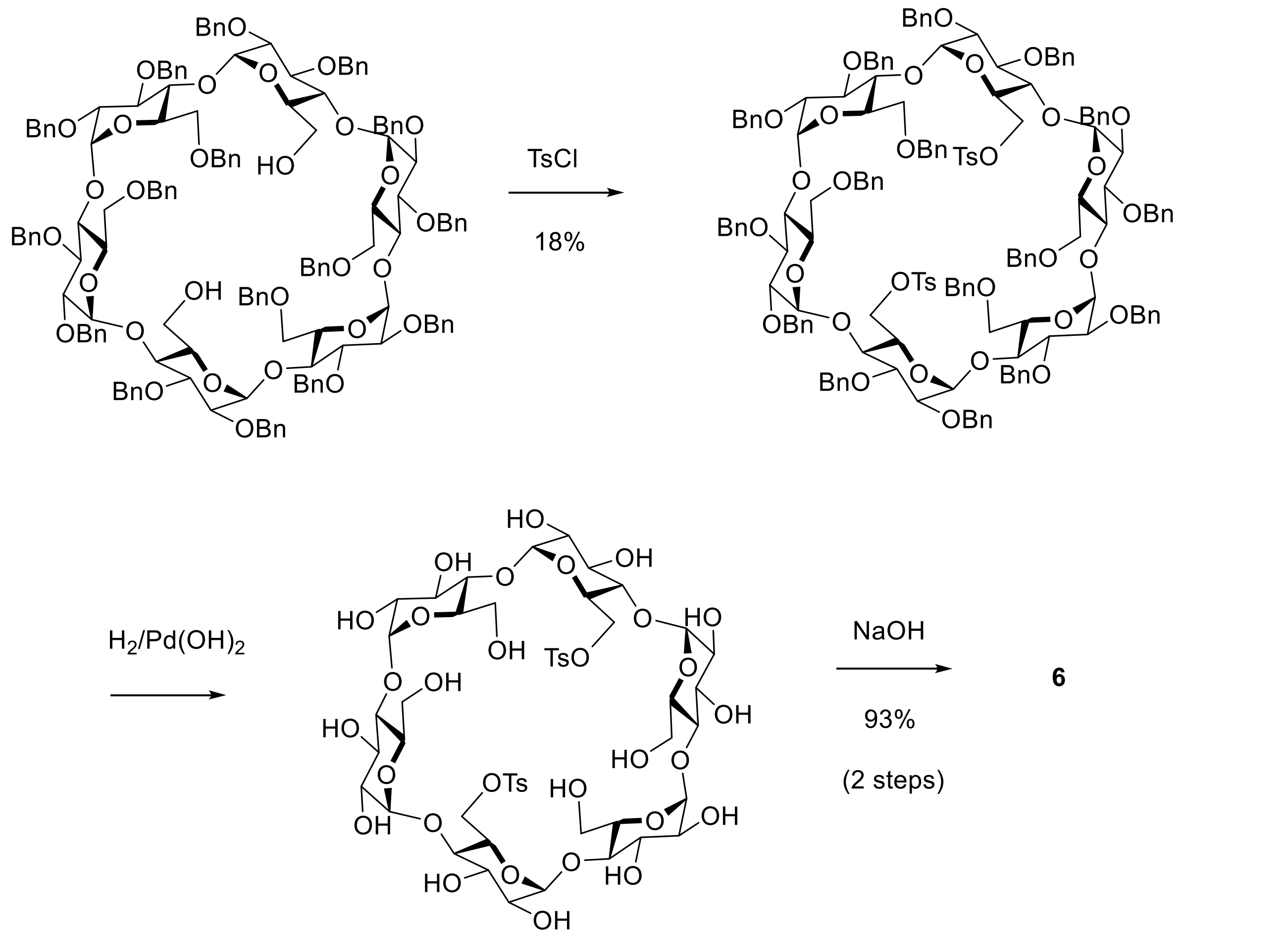

Synthesis of 3^{A},6^{A};3^{D},6^{D}-dianhydro-α-cyclodextrin (6). 2^{A-F},3^{A-F},6$^{C-D,E-F}$-hexadeca-*O*-benzyl-*α*-cyclodextrin[1,2] (4.58 g, 1.90 mmol) was dissolved dry pyridine (70 mL) followed by addition of *p*-toluenesulfonyl chloride (3.66 g, 19.2 mmol). The reaction mixture was stirred at room temperature under a nitrogen atmosphere. The reaction was run for 3 days until full conversion of starting material, as indicated by TLC (2:1 Heptane/EtOAc). The reaction was quenched with water (70 mL) and DCM (70 mL). The aqueous phase was extracted with DCM (2 × 100 mL) and the combined organic phases were washed with water (200 ml), 6 M HCl (200 mL), 1 M (100 mL) and finally with $NaHCO_3$ (2 × 250 mL). Evaporation of the solvent *in vacuo* resulted in a yellow-brownish foam. The crude product was purified by automated flash column chromatography (heptane/EtOAc, 0–100% EtOAc) to yield in fractions 13-15 the 6^{A}-chlorodeoxy-6^{D}-O-tosylate (670 mg) and in fractions 18–20 the ditosylate (950 mg, 0.349 mmol, 18%).

The ditosylate (2^{A-F},3^{A-F},6$^{B-C,E-F}$-hexadeca-*O*-benzyl-6^{A,D}-di-*O*-(*p*-toluenesulfonyl)-*α*-cyclodextrin, 950 mg, 0.349 mmol) was dissolved in a mixture of MeOH (50 mL) and EtOAc) (50 mL) . After 5 h 1 M HCl (1 drop) was added to the reaction mixture. The reaction mixture was left to stir under a hydrogen atmosphere in the presence of Pearlman's catalyst/$Pd(OH)_2$/C (101 mg, 0.719 mmol/719 μmol). After 16 h TLC analysis showed almost full conversion of starting material and water (10 mL) was added to prevent precipitation of the product. After 5 h another charge of Pearlman's catalyst (42 mg, 0.302 mmol) was added and the reaction

was stirred for another 20 h. Filtration of the reaction mixture through a pad of celite and removal of solvents *in vacuo* resulted in a white powder that was purified by automated flash column chromatography (MeCN/MeOH, 0–100% MeOH) to yield the desired 6^A,6^D-di-*O*-tosyl-α-cyclodextrin (0.2 g). ^{1}H NMR (500 MHz, $CDCl_3$) δH: 7.79 (d, *J* = 8.3 Hz, 2H, CHTs), 7.44 (d, *J* = 8.2 Hz, 2H, CHTs), 4.97 – 4.91 (m, 6H, H-1), 4.40 (dd, *J* = 10.9, 1.9 Hz, 2H, H-6a^A), 4.27 (dd, *J* = 10.9, 5.5 Hz, 2H, H-6b^A), 4.03 (ddd, *J* = 10.1, 5.5, 1.9 Hz, 2H, H-5Ts), 3.97 – 3.73 (m, 14 H, H-3, H-5, H-6), 3.71 –3.64 (m, 4H, H-6), 3.58 – 3.45 (m, 8H, H-4, H-2), 3.41 – 3.36 (m, 4H, H-2, H-4) ppm. ^{13}C-NMR (126 MHz, $CDCl_3$) δ: 146.6, 134.3 (*i*-CTs), 131.1, 129.1 (CHTs), 103.8, 103.6, 103.1 (C-1), 83.2, 83.1, 82.8, 75.1, 75.0, 75.0, 73.8, 73.7, 73.6, 73.5 (C-2, C-3, C-4, C-5), 71.1 (C-5Ts), 70.6 (C-6Ts), 61.7, 61.5 (C-6), 21.6 (CH_3 Ts) ppm.

6^A,6^D-Di-*O*-(*p*-toluenesulfonyl)-α-cyclodextrin (199 mg, 155 μmol) was dissolved in 1 M NaOH (2.3 mL) and stirred at 40 ℃ for 23 h. The solution was neutralized with the addition of 1 M HCl followed by filtration and evaporation of solvent in vacuo to yield the crude product as a white powder. The crude product was purified by mixed bead ion chromatography, Dowex Marathon MR-3 (12 mL, H_2O) to yield the desired product 3^A,6^A;3^D,6^D-dianhydro-α-cyclodextrin (**6**) as a white foam (135 mg, 144.0 μmol, 93%).

HRMS (MALDI+): Calculated for $C_{36}H_{56}O_{28}Na^+$ ($M + Na$) m/z: 959.28503, found: 959.28351. NMR was assigned using COSY, Roesy, Tocsy and HSQC:

^{1}H NMR (800 MHz, D_2O) δH: 5.18 (d, *J* = 3.0 Hz, 2H, H-1^{A,D}), 5.12 (d, *J* = 3.8 Hz, 2H, H-1^{C,F}), 5.07 (d, *J* = 3.8 Hz, 2H, H-1^{B,E}), 4.64 (app. t, *J* = 2.7 Hz, 2H, H-5^{A,D}), 4.53 (t, *J* = 5.0 Hz, 2H, H-3^{A,D}), 4.31 (d, *J* = 11.0 Hz, 2H, H-6a^{A,D}), 4.24 (dt, *J* = 10.1, 2.4 Hz, 2H, H-5^{B,E}), 4.16 (dd, *J* = 5.5, 2.6 Hz, 2H, H-4^{A,D}), 4.05 (dd, *J* = 11.0, 3.0 Hz, 2H, H-6b^{A,D}), 4.03 – 3.99 (m, 4H, H-2^{A,D}, H-6a^{B,E}), 3.96 –3.92 (m, 4H, H-3^{B,E},H-6a^{C,F}), 3.85 (dd, *J* = 12.3, 2.2 Hz, 2H, H-6b^{B,E}), 3.83 – 3.78 (m, 4H, H-5^{C,F}, H-6b^{C,F}), 3.76 (t, *J* =9.5 Hz, 2H, H-3^{C,F}), 3.72 (t, *J* = 9.6 Hz, 2H, H-4^{B,E}), 3.68 (t, *J* = 9.6 Hz, 2H, H-4^{C,F}), 3.66 (dd, *J* = 10.2, 3.8 Hz, 2H, H-2^{B,E}), 3.61 (dd, *J* = 9.8, 3.7 Hz, 2H, H-2^{C,F}) ppm.

^{13}C NMR (201 MHz, D_2O) δC: 102.2 (C-1^{C,F}), 100.9 (C-1^{A,D}), 100.8 (C-1^{B,E}), 81.1, 81.0 (C-4^{B,C,E,F}), 79.2 (C-4^{A,D}), 74.0 (C-5^{A,D}), 72.7 (C-3^{B,E}), 72.6 (C-3^{C,F}), 72.5 (C-2^{C,F}), 71.8 (C-3^{A,D}), 71.5 (C-5^{C,F}), 71.3 (C-5^{B,E}), 70.9 (C-2^{B,E}), 69.2 (C-6^{A,D}) , 68.8(C-2^{A,D}) , 59.9 (C-6^{C,F}), 59.7 (C-6^{B,E}) ppm.

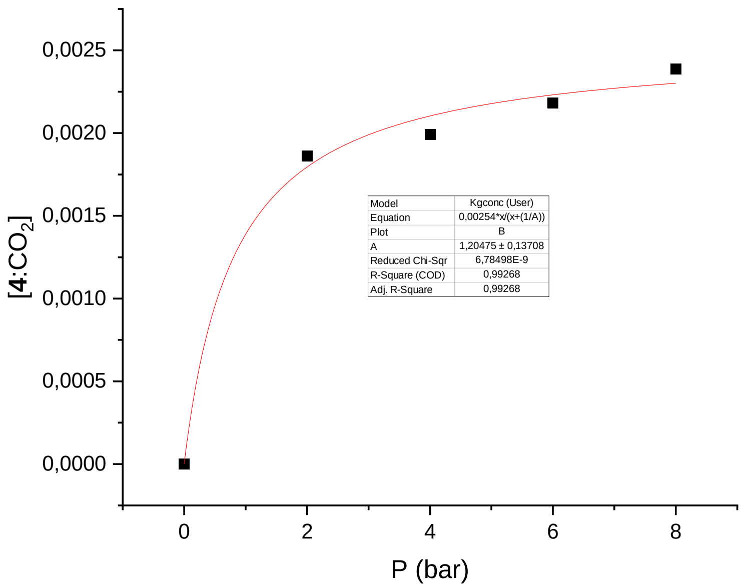

Figure S1. Plot of **4**(CO_2) complex formation as a result of increasing pressure of CO_2.

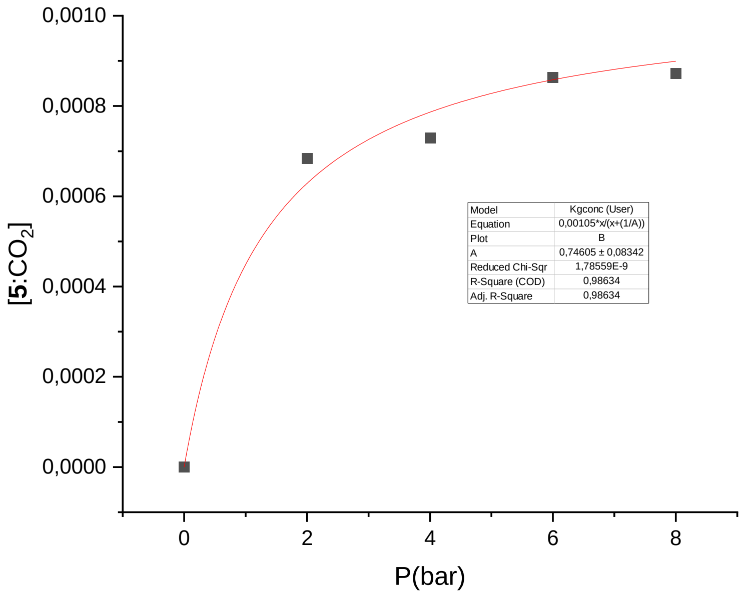

Figure S2. Plot of **5**(CO_2) complex formation as a result of increasing pressure of CO_2.

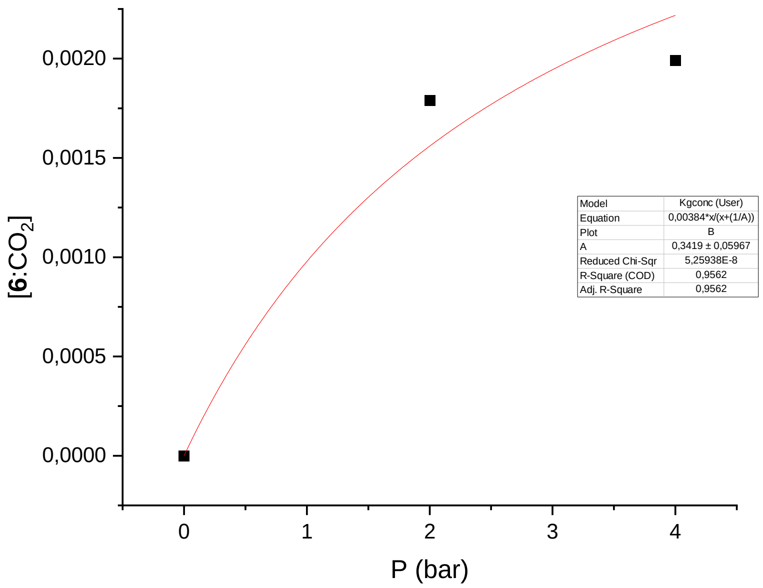

Figure S3. Plot of **6**(CO_2) complex formation as a result of increasing pressure of CO_2.

Design of pressure cell

The pressure cell was build by the Niels Bohr Institute Technical Support group according to the following scheme:

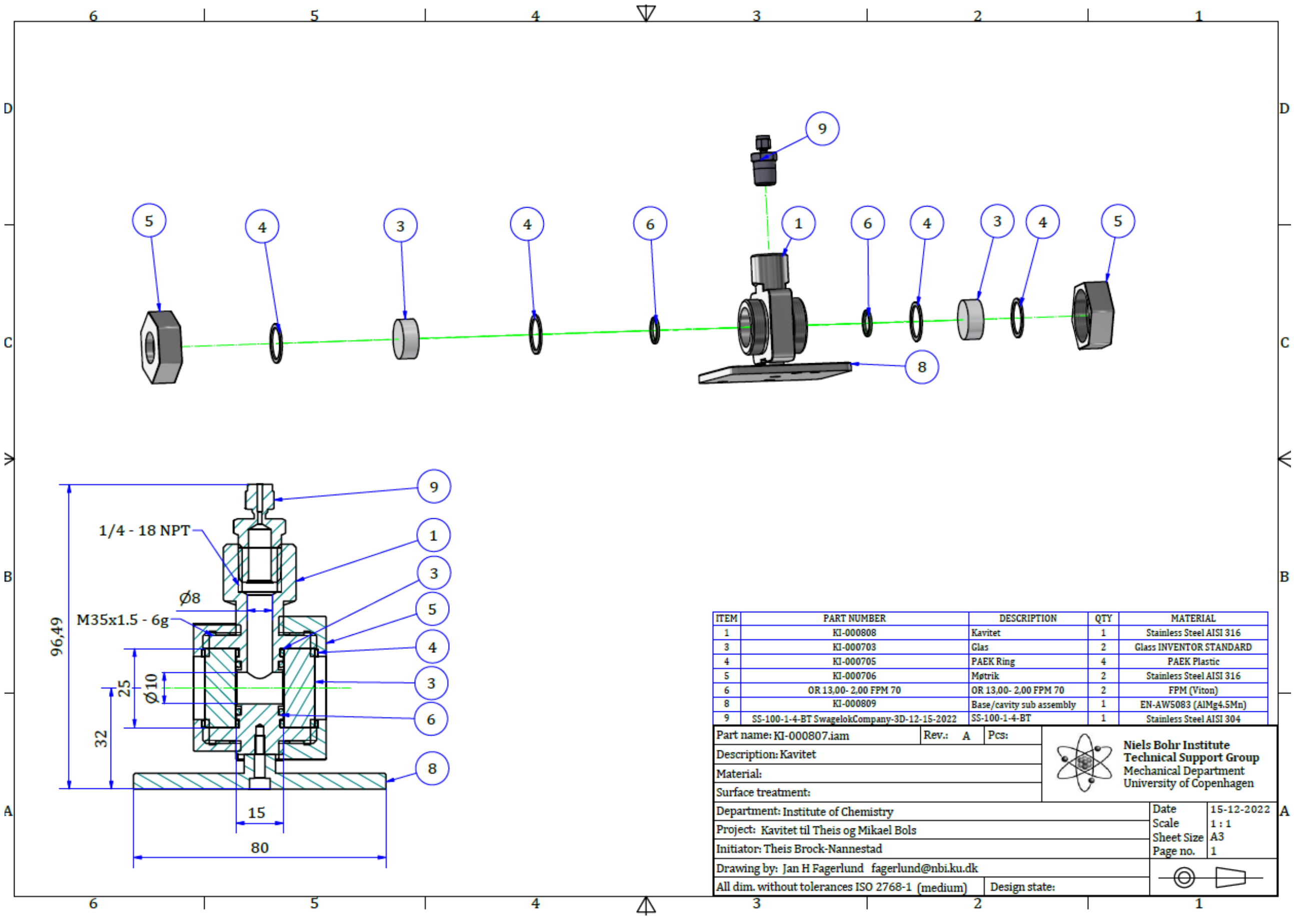

References

(1) Lecourt, T.; Herault, A.; Pearce, A. J.; Sollogoub, M.; Sinay, Pierre. *Chemistry - A European Journal* **2004**, *10*, 2960–2971. doi:10.1002/chem.200305683

(2) Pearce, A. J.; Sinay, Pierre. *Angewandte Chemie, International Edition* **2000**, *39*, 3610–3612

^{1}H NMR (800 MHz; D_2O) of **5**

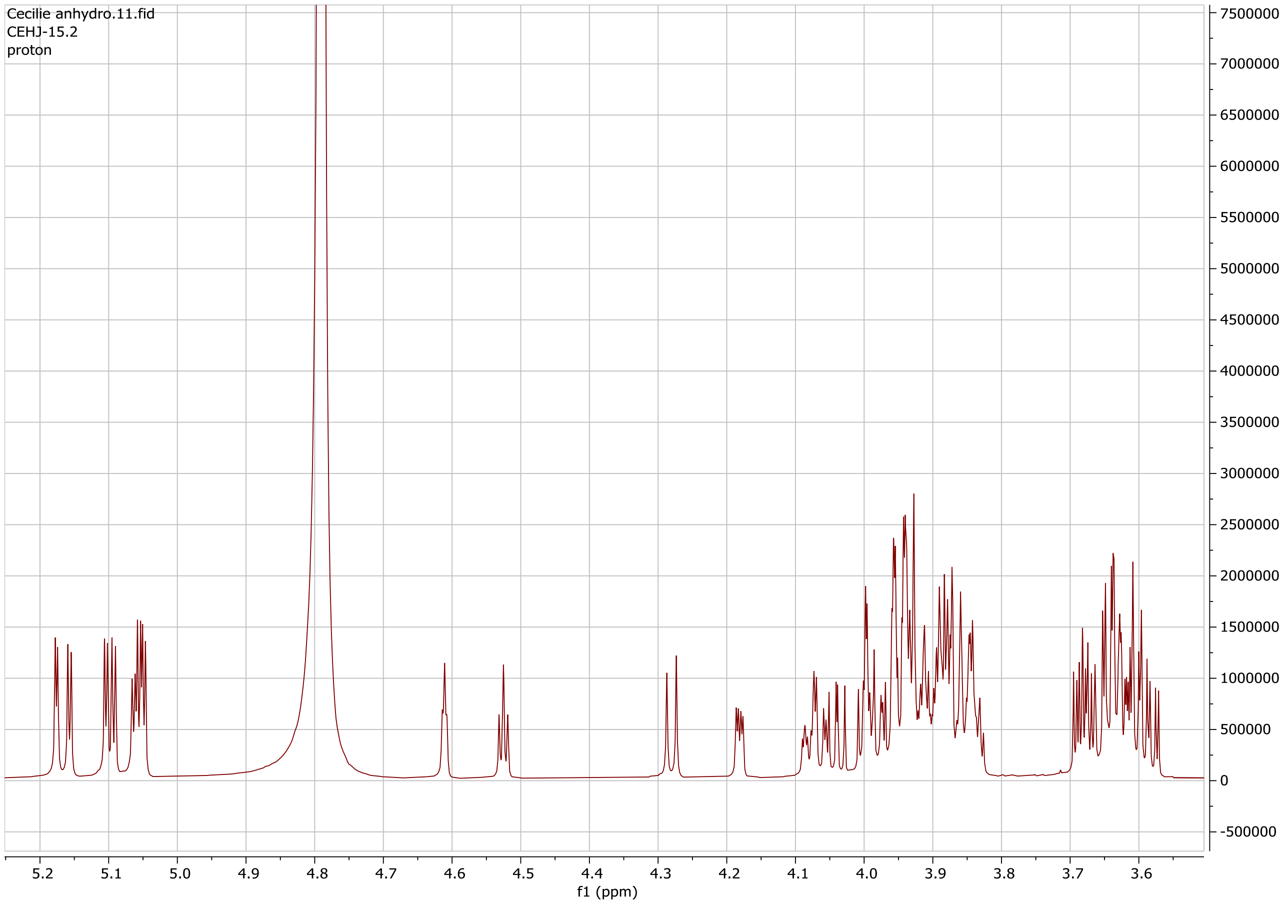

^{13}C NMR (201 MHz; D_2O) of **5**

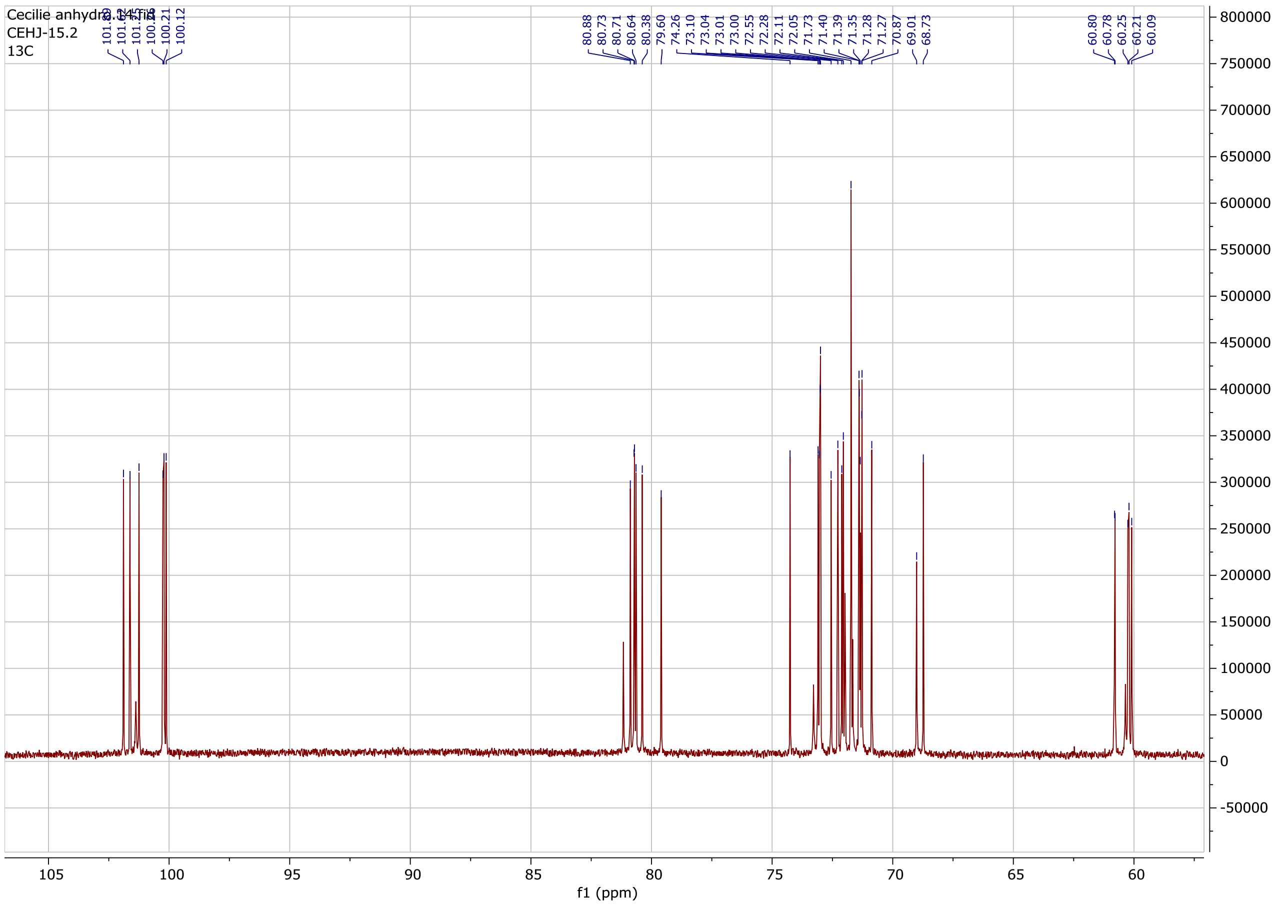

Cosy (800 MHz; D_2O) of **5**

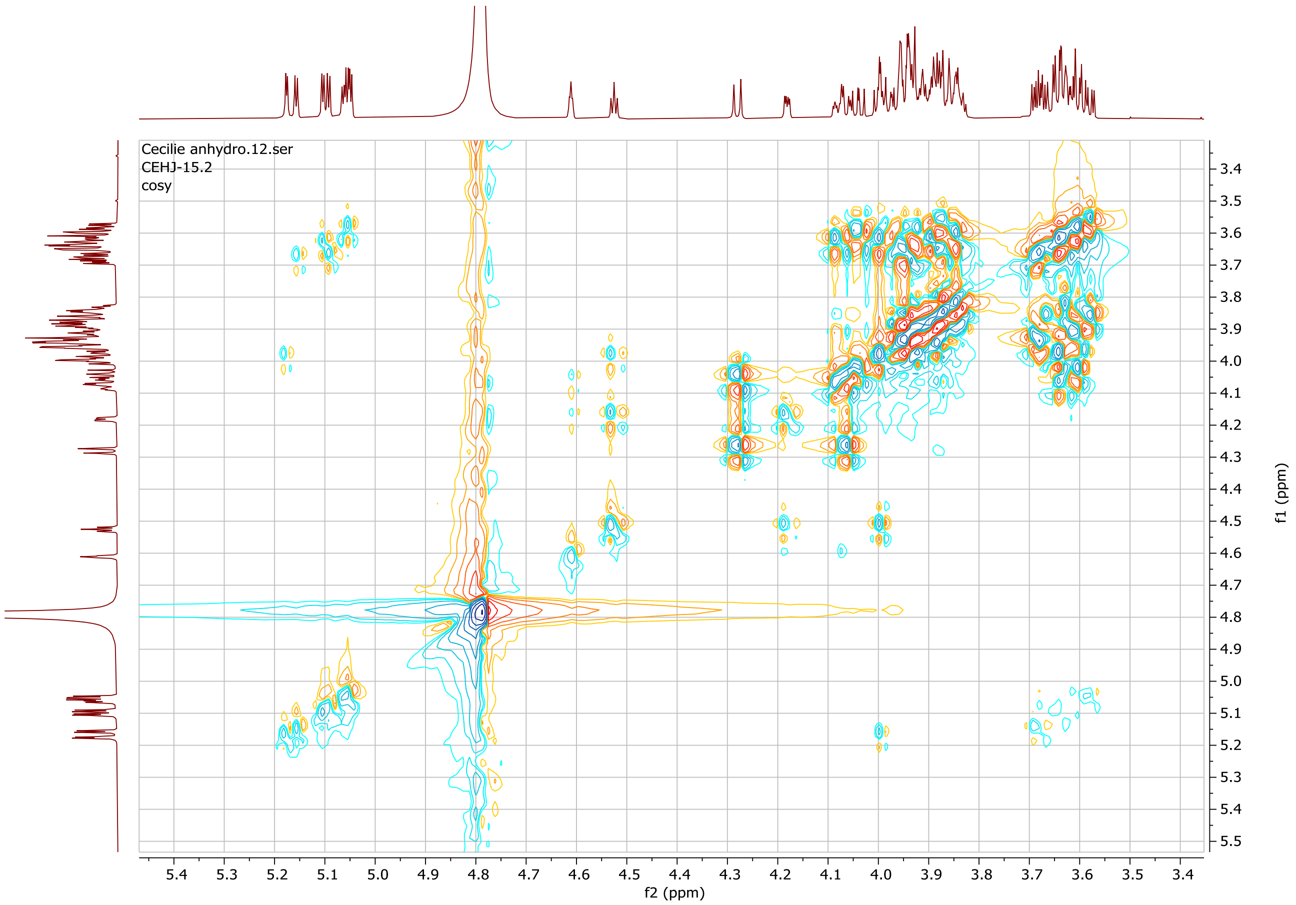

HSQC (800 MHz; D_2O) of **5**

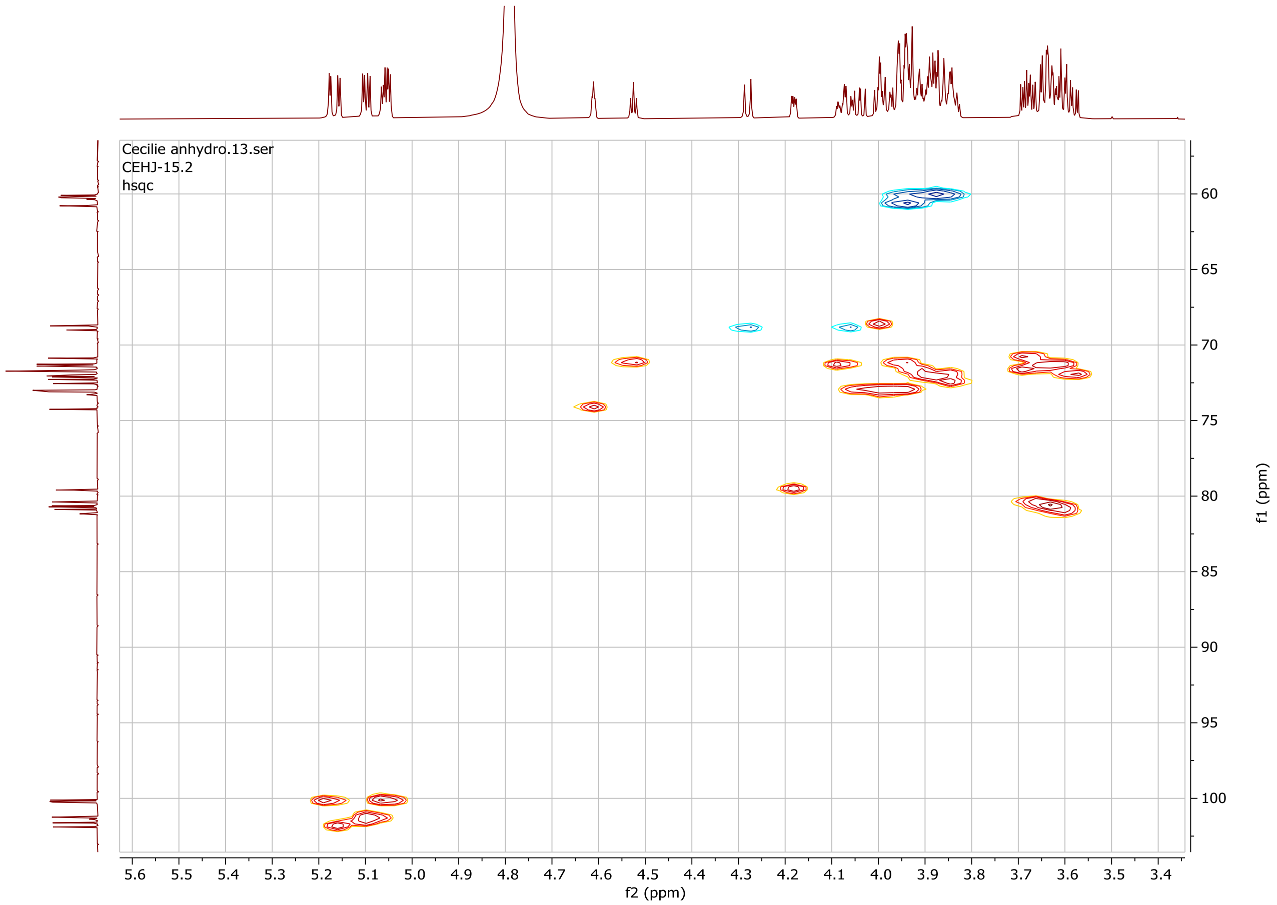

HSQC-Tocsy (800 MHz; D_2O) of **5**

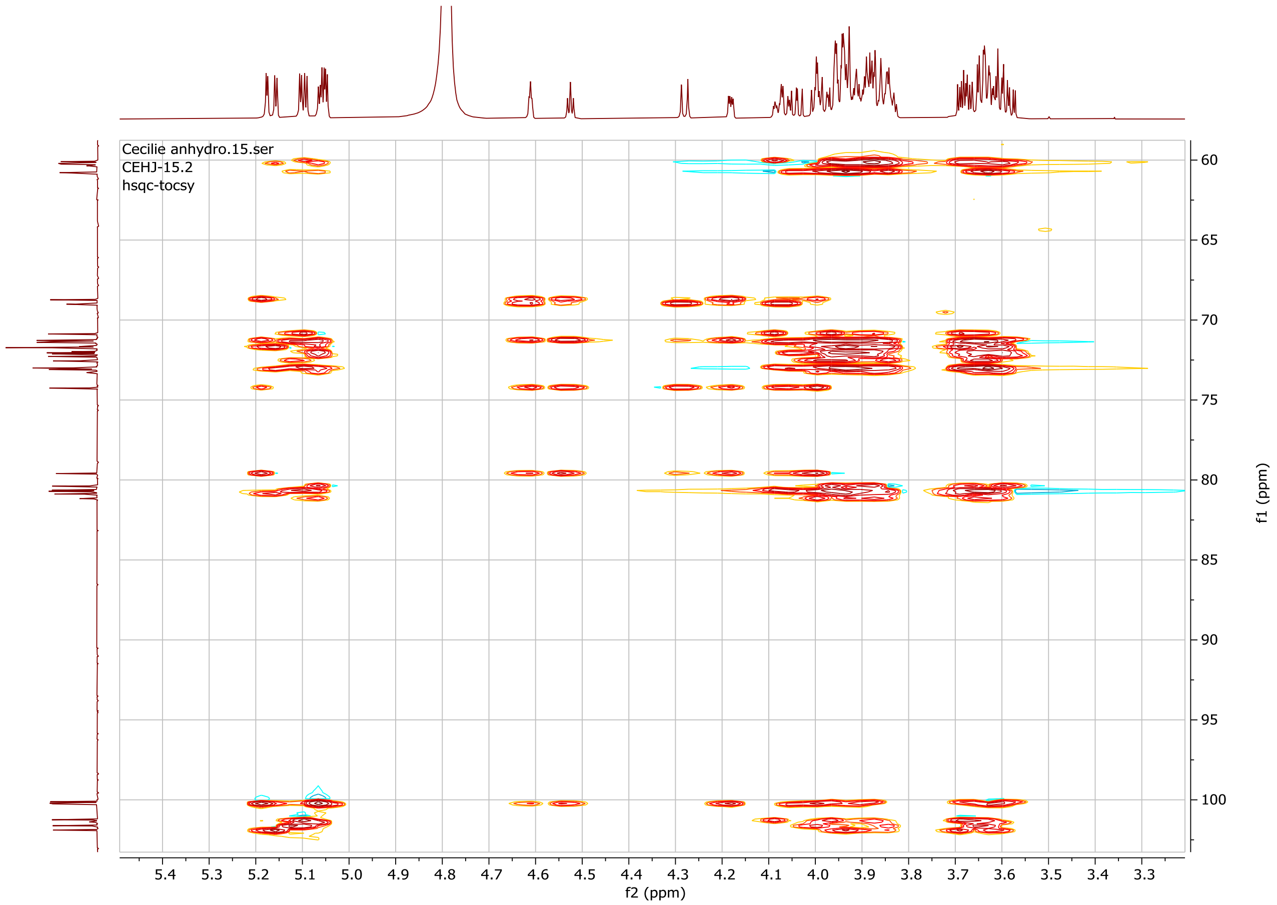

Roesy (800 MHz; D_2O) of **5**

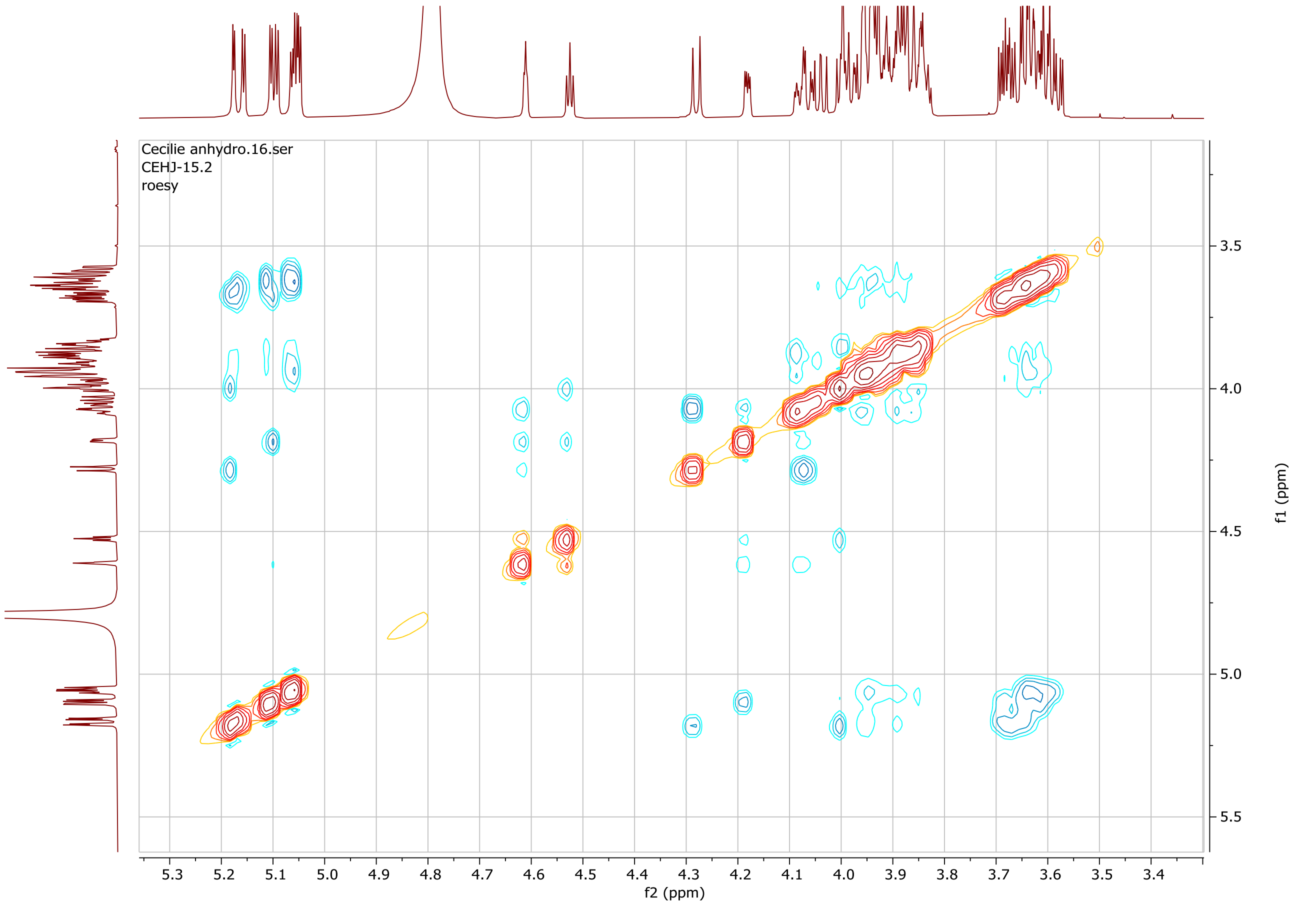

^{1}H NMR (800 MHz; D_2O) of **6**

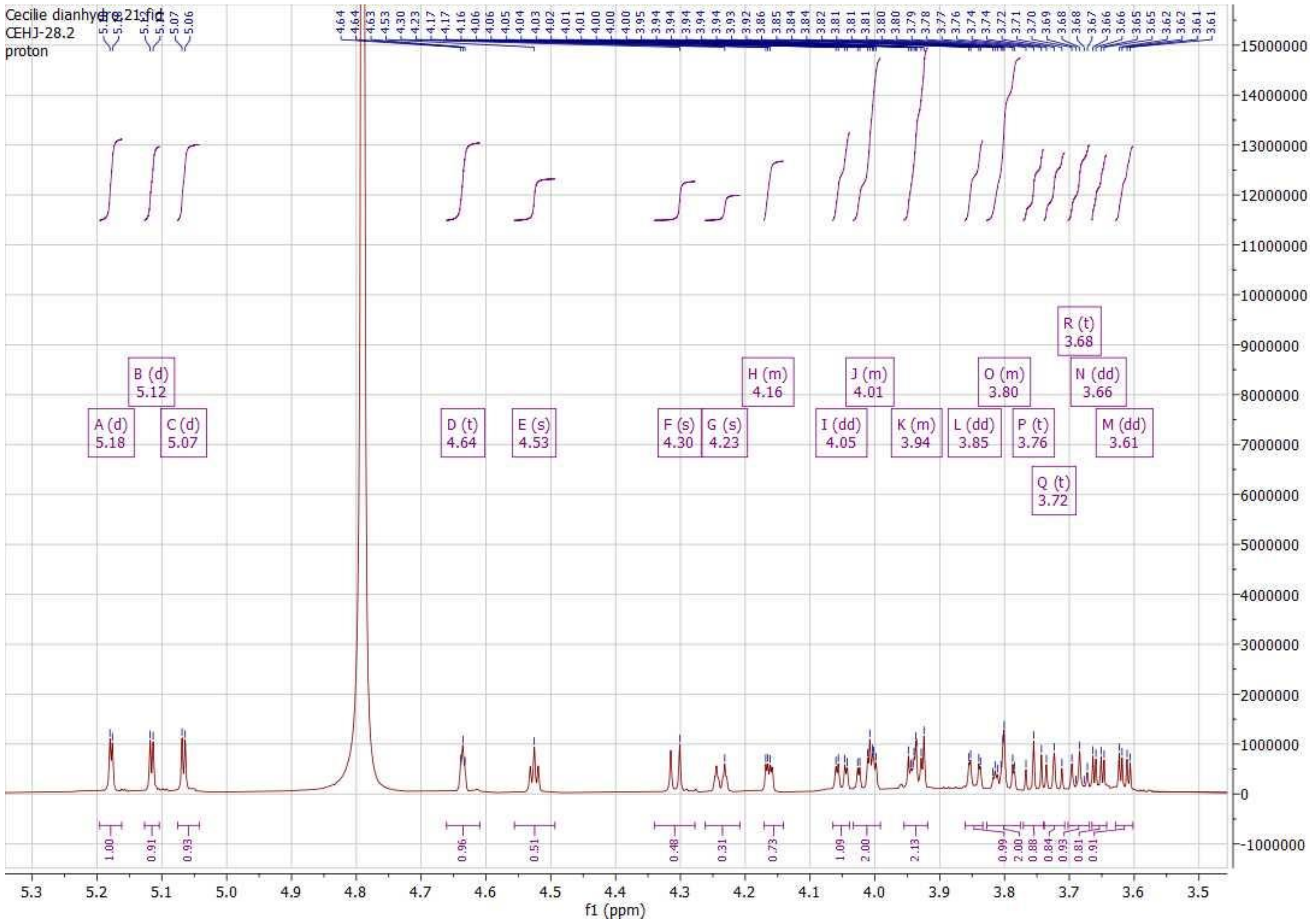

^{13}C NMR (201 MHz; D_2O) of **6**

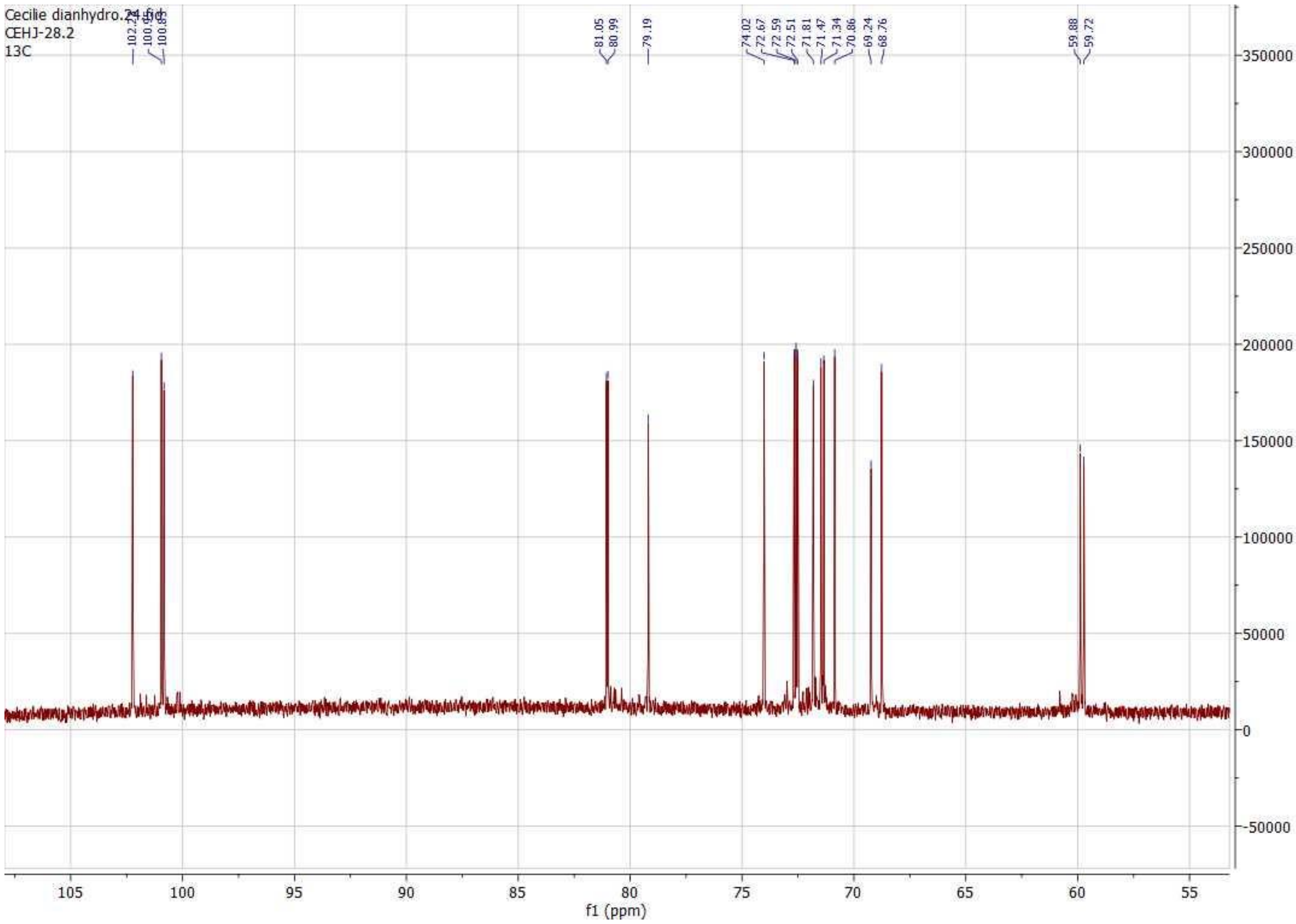

Cosy (800 MHz; D_2O) of **6**

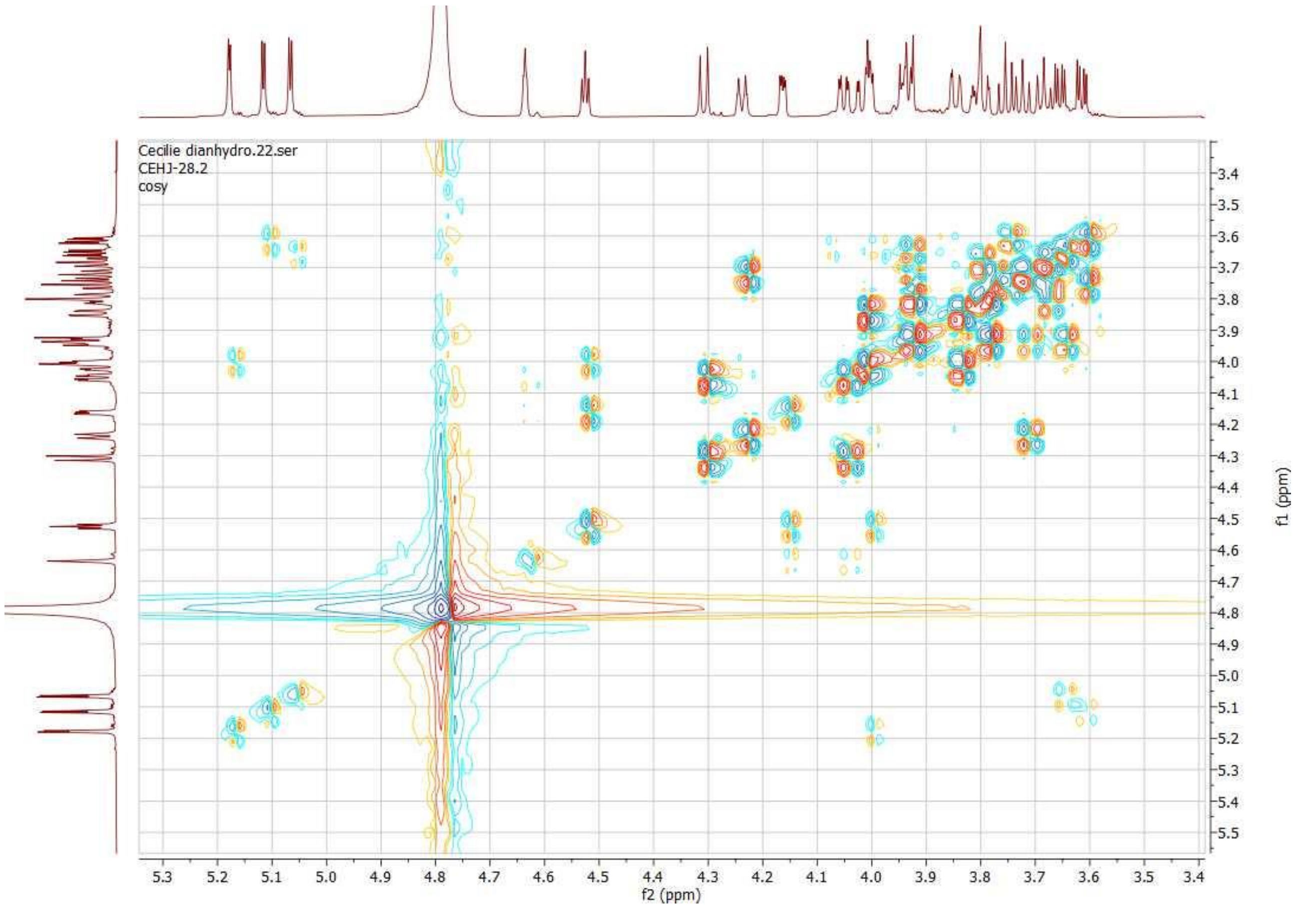

HSQC (800 MHz; D_2O) of **6**

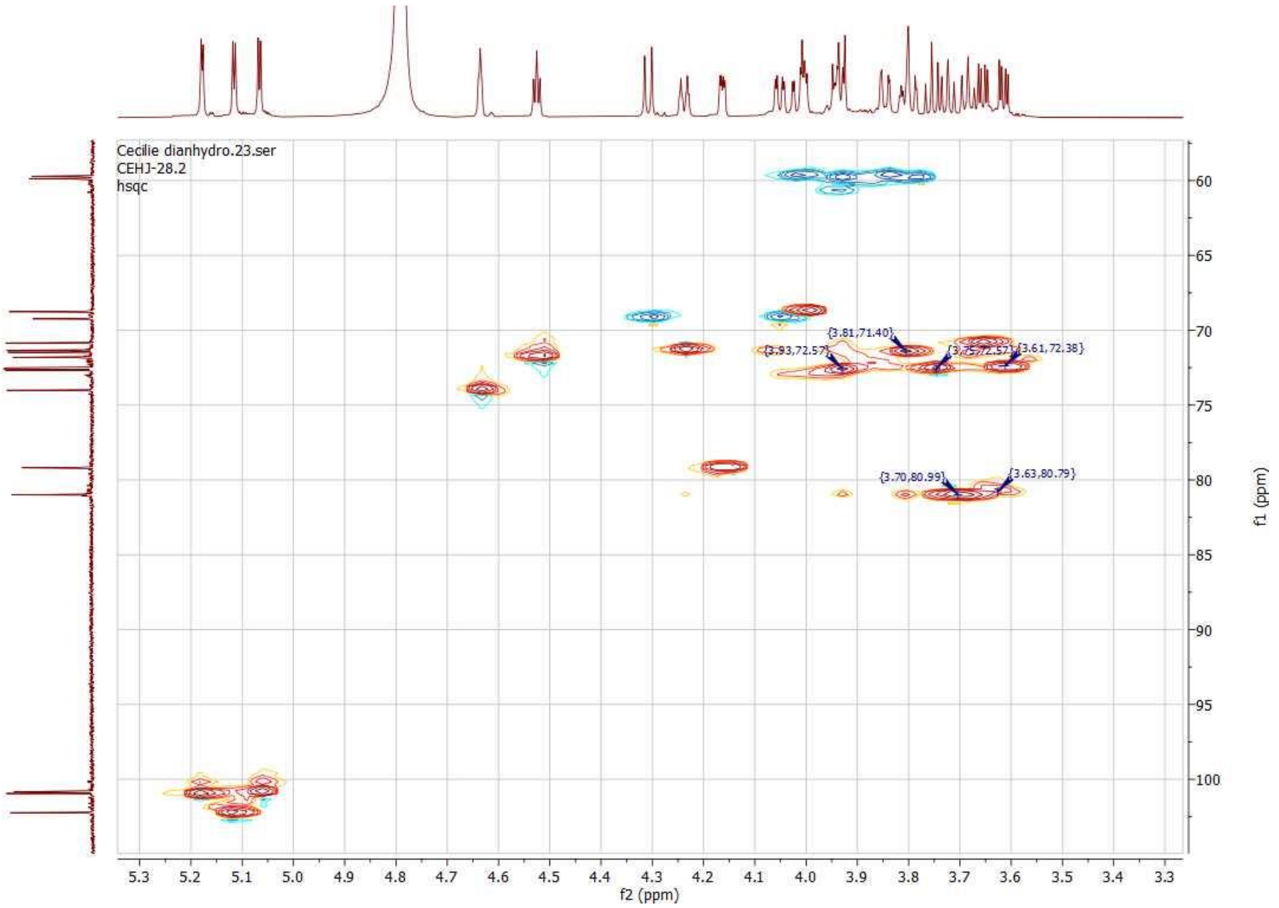

HSQC-Tocsy (800 MHz; D_2O) of **6**

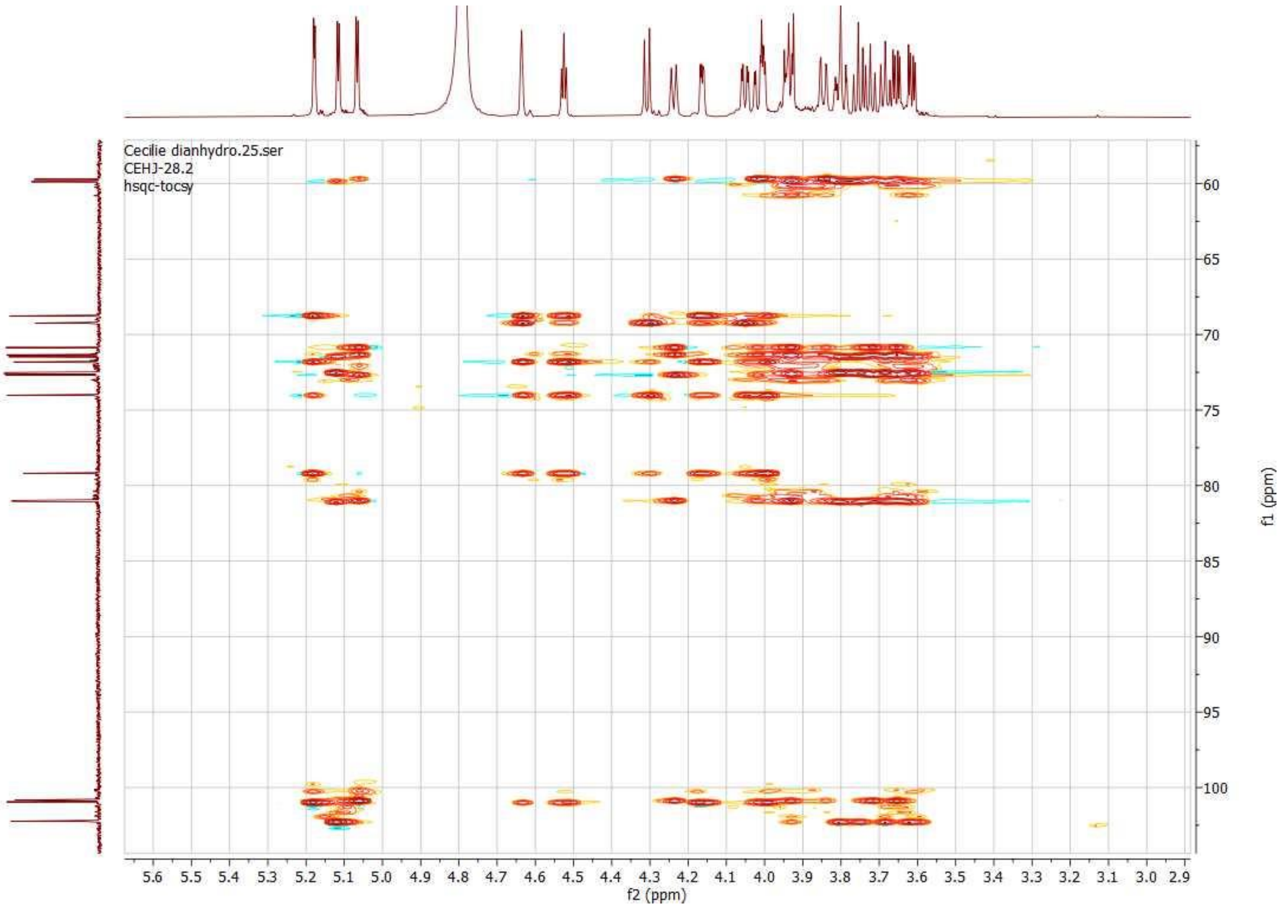

Roesy (800 MHz; D_2O) of **6**

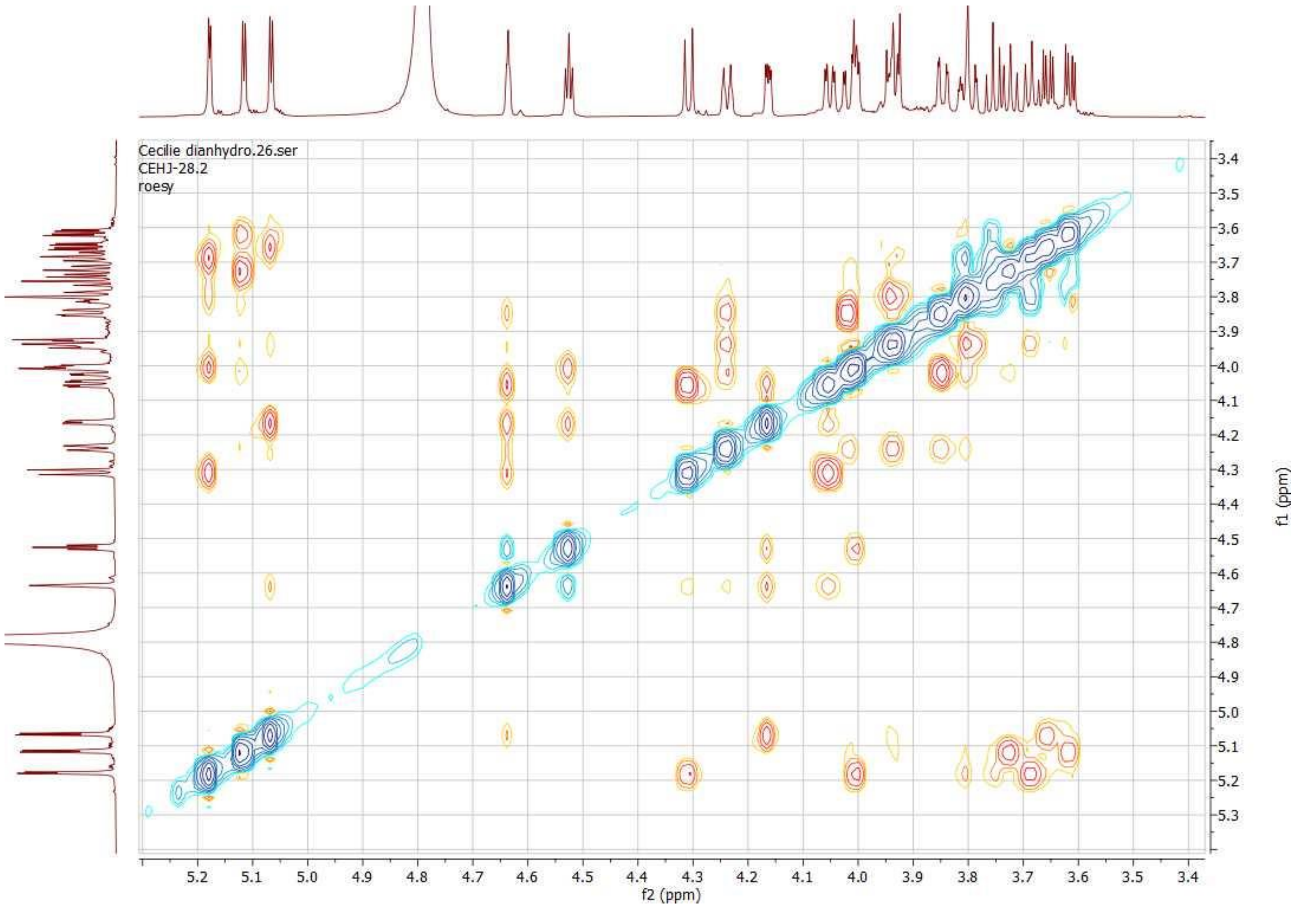

Supplement: File 1 — Experimental and analytical data. [file Beilstein_J_Org_Chem-19-1021-s001.pdf]
